# Dealing with the Pandemic and After for Children and Adolescents, and their Families

**DOI:** 10.1192/j.eurpsy.2022.53

**Published:** 2022-09-01

**Authors:** G. Apter

**Affiliations:** Groupe Hospitalier du Havre, Service Universitaire Havrais De Psychiatrie Périnatale Et De L’enfant, LE HAVRE Cédex, France

## Abstract

France has been severely impacted by the pandemic. The first wave imposed a major lockdown, never seen before. The second and third wave lockdown responses were somewhat less dramatic. People were allowed to travel to work. Nursery and elementary schools were left open while high schools had part-time attendance. Spring 2021, still presented with a terrible death toll of 300/day, for a 67million population country. For many months the spotlights were focused on the high levels of mortality and morbidity of the elderly. This somehow obliterated the younger generation’s mental health issues. It now appears that children and adolescents have had to pay a steep price to Covid 19. In France, during the first lockdown, child and adolescent morbidity and mortality due to abuse heightened considerably compared to 2019 during the same period. And during the second and mostly the third lockdown, pediatric emergency wards have been underwater with youth mental health issues ranging form suicidal ideation to acute dissociation. Different hypotheses have emerged on how the pandemic has so dramatically impacted the mental health of children and adolescents, specifically the most vulnerable ones. The consequences this might have for child development and mental health during the years to come will be debated.

**Disclosure:**

No significant relationships.

